# Children Living with HIV-Infected Adults: Estimates for 23 Countries in sub-Saharan Africa

**DOI:** 10.1371/journal.pone.0142580

**Published:** 2015-11-17

**Authors:** Susan E. Short, Rachel E. Goldberg

**Affiliations:** 1 Department of Sociology and Population Studies and Training Center, Brown University, Providence, RI, United States of America; 2 Department of Sociology, University of California Irvine, Irvine, CA, United States of America; Tulane University School of Public Health, UNITED STATES

## Abstract

**Background:**

In sub-Saharan Africa many children live in extreme poverty and experience a burden of illness and disease that is disproportionately high. The emergence of HIV and AIDS has only exacerbated long-standing challenges to improving children’s health in the region, with recent cohorts experiencing pediatric AIDS and high levels of orphan status, situations which are monitored globally and receive much policy and research attention. Children’s health, however, can be affected also by living with HIV-infected adults, through associated exposure to infectious diseases and the diversion of household resources away from them. While long recognized, far less research has focused on characterizing this distinct and vulnerable population of HIV-affected children.

**Methods:**

Using Demographic and Health Survey data from 23 countries collected between 2003 and 2011, we estimate the percentage of children living in a household with at least one HIV-infected adult. We assess overlaps with orphan status and investigate the relationship between children and the adults who are infected in their households.

**Results:**

The population of children living in a household with at least one HIV-infected adult is substantial where HIV prevalence is high; in Southern Africa, the percentage exceeded 10% in all countries and reached as high as 36%. This population is largely distinct from the orphan population. Among children living in households with tested, HIV-infected adults, most live with parents, often mothers, who are infected; nonetheless, in most countries over 20% live in households with at least one infected adult who is not a parent.

**Conclusion:**

Until new infections contract significantly, improvements in HIV/AIDS treatment suggest that the population of children living with HIV-infected adults will remain substantial. It is vital to on-going efforts to reduce childhood morbidity and mortality to consider whether current care and outreach sufficiently address the distinct vulnerabilities of these children.

## Introduction

In sub-Saharan Africa many children live in extreme poverty and experience a burden of illness and disease that is disproportionately high [1, 2]. Despite declines, the under-five mortality rate in the region is still 16 times that of developed countries, and children continue to die from preventable or treatable causes, including infectious diseases such as pneumonia, diarrhea, or malaria [2]. The emergence of HIV and AIDS several decades ago, which disproportionately affects sub-Saharan Africa, has only exacerbated long-standing challenges to improving children’s health. In 2013, 25 million adults and children lived with HIV in sub-Saharan Africa—71% of the total infected global population [3].

The importance of this HIV context for children’s vulnerabilities has been recognized by scholars, practitioners, and policymakers [4]. The Global Plan towards the elimination of new infections among children has emphasized reducing mother-to-child transmission, and between 2009 and 2013, the number of new HIV infections in children has been reduced by 43% in the designated 21 priority countries in sub-Saharan Africa [3], though many children remain undiagnosed [5]. Aside from pediatric infection, vulnerabilities result from living in families and communities deeply affected by HIV. A particularly visible consequence has been a rise in the number of orphans in the region. While not all parental deaths result from AIDS, in 2013 over 15 million children in sub-Saharan Africa were estimated to have lost one or both parents to AIDS [4]. In Swaziland and Lesotho, the countries with the highest HIV prevalence, an estimated 70% of orphans lost their parents to AIDS [1]. Children who experience parental death have been found to be at increased risk of poorer health and schooling outcomes relative to other children [6–11].

Even in the absence of pediatric infection or adult mortality, living in households or families with HIV-infected adults can render children vulnerable. Children living in close proximity to infected adults face exposure to opportunistic infections (OIs) like tuberculosis, hepatitis, pneumonia, and diarrheal disease [12]. While the introduction of antiretroviral therapy (ART) has contributed to a marked improvement in the quality of life and OI mortality of HIV-infected persons, risks of OIs persist because individuals may not know their HIV status, patients may not be on needed ART, or patients on ART may have poor adherence [12]. While all co-resident household members may be at increased risk of exposure to OIs, young children may be especially susceptible because they experience the most direct contact with adults, spend much of their time in the home, and upon exposure, can experience more severe illness than older household members. In addition, children’s symptoms, such as respiratory symptoms associated with TB or pneumonia, can be mistakenly attributed to less serious common childhood illnesses, and not treated [13]. Older children are also at risk, particularly because it is not uncommon for them to provide care for ill adults [14].

Besides direct exposure to adult illness, the health and well-being of children living in a household with an HIV-infected adult can be compromised if attention of household adults or older children is redirected toward the infected adult; children’s household responsibilities increase; or resources needed for schooling, nutrition, or medical care are diverted away from the child [15–18]. This situation may be compounded by financial strain associated with a change in the work status of an ill adult. In addition, children sometimes experience HIV-associated stigma in their communities and schools, and among networks of family and friends, which may negatively affect their health and well-being, including mental health [19, 20].

Several strands of emerging research in the region provide evidence of diminished outcomes for children living with and caring for HIV-infected adults. Single and multi-country studies link having an HIV-infected parent with malnutrition, lack of medical care, excess infant and childhood mortality risk, and decreased likelihood of school attendance [21–25]. Research also reveals higher burdens of acute and chronic morbidity for children whose parents have an AIDS-related illness [17], and elevated risk of pulmonary tuberculosis symptoms for children who provide care for co-resident ill adults [13, 14, 26].

In this article, we document the extent to which children in sub-Saharan Africa live in households with HIV-infected adults. Despite growing recognition that children living with HIV-infected adults are also HIV-affected, most publications that monitor the situation of children in the context of AIDS report only the prevalence and incidence of pediatric HIV, the percentage of HIV-infected pregnant women receiving treatment, and the prevalence of children who have lost one or both parents to AIDS or other causes [4, 27]. A notable exception is a Demographic and Health Survey (DHS) report that assessed the situation of orphans and vulnerable children across eight sub-Saharan African countries, and included a measure of the proportion of children living in households with an HIV-infected adult [28]. In this study, we estimate the prevalence of children living in households with HIV-infected adults based on recent DHS data from 23 countries in the region. We also characterize this population of children further by assessing its overlaps with the orphan population, as a child whose parent has died from complications of AIDS may also be likely to live with an HIV-infected adult if he or she lives with a surviving parent. In addition, because it is quite common for children in the region to be co-resident with non-parental adults [29–34], we document how frequently the HIV-infected adults in children’s households are their mothers or fathers. Since interventions targeted at children in families involve outreach to adults, additional detail on the nature of the relationship between the children and the HIV-infected adults in their households may be useful for focusing multi-sector response.

Measuring the population of children living in households with HIV-infected adults is critical and timely. Global efforts to encourage HIV testing and treatment have resulted in substantial increases in the number of people receiving treatment [35]. The United Nations reports that there was a 20-fold increase in the uptake of antiretroviral treatment (ART) in the developing world between 2003 and 2011 [36]. In sub-Saharan Africa, 7.5 million people received ART for HIV or AIDS in 2012 [37], though in 2013, treatment coverage was estimated to be at only 37% of all people living with HIV [3]. As treatment grows, and more adults learn their status and receive treatment, parents and other adult household members who in past years might have died of AIDS-related illnesses can be expected to live longer. Programmatic news on efforts to reduce HIV infections is also positive, but new infections are still far too common. In 2013, 1.5 million people were newly infected with HIV in sub-Saharan Africa [38]; efforts to reduce infections among adults have been far less successful than efforts to reduce infections among children [3]. The combination of reductions in HIV mortality due to increased treatment, sustained population growth, and the persistent addition of newly-infected individuals who are more frequently adults and less frequently children, suggests that the population of children living in households with HIV-infected adults, now and in the near future, will be substantial [39].

## Methods

### Ethical Considerations

All analyses conducted in this paper are based on secondary data with all participant identifiers removed. The ICF International Institutional Review Board (IRB), which requires compliance with the U.S. Department of Health and Human Services regulations for the protection of human subjects (45 CFR 46), reviewed and approved all procedures and questionnaires. In addition, typically an IRB in the host country ensured that the survey complied with laws and norms of the nation. Ethical permission to use the data was obtained from ORC Macro Inc. Additional detail on the procedures regarding the protection of human subjects is available on the DHS website (http://dhsprogram.com/What-We-Do/Protecting-the-Privacy-of-DHS-Survey-Respondents.cfm#sthash.YAZ3GK1r.dpuf).

### Data and Analyses

Data are from nationally representative population samples collected as part of the Demographic and Health Surveys. The DHS use standardized questionnaires and protocols to facilitate cross-country analyses. Since 2001, many DHS surveys have included HIV testing. We include in our analysis all DHS surveys conducted in sub-Saharan Africa that include HIV testing as part of the Standard DHS or AIDS Indicator Surveys (AIS). In addition, we limit the sample to surveys that possess the information required to link children with their biological parents and ascertain parents’ survival status. In the case of a country with repeated surveys that fit these criteria, results from the most recent survey are reported. Our final sample includes data for 23 countries collected between 2003 and 2011. Background detail, including characteristics of the sampled populations, DHS/AIS testing rates, and adult HIV prevalence, are summarized in Table 1.

**Table 1:** Sample Characteristics and Adult HIV Prevalence.

Country	Survey year	Total N	Age range of women tested for HIV	Percent of eligible women tested*	Age range of men tested for HIV	Percent of eligible men tested*	Adult HIV prevalence (15–49)**
***Southern Africa***							
Lesotho	2009	9,987	15–49	93.6	15–59	88.0	23.0
Malawi	2010	22,788	15–49	90.5	15–54	83.7	10.6
Mozambique	2009	14,028	15–64	92.3	15–64	91.7	11.5
Swaziland	2007	11,190	15–49	87.2	15–49	77.6	25.9
Zambia	2007	19,462	15–49	77.1	15–59	72.2	14.3
Zimbabwe	2011	20,458	15–49	79.9	15–54	69.3	15.2
***Central and East Africa***							
Cameroon	2011	17,864	15–49	93.7	15–59	92.3	4.3
DRC	2007	12,799	15–49	90.3	15–59	86.3	1.3
Ethiopia	2011	39,538	15–49	89.3	15–59	81.8	1.5
Kenya	2009	9,615	15–49	86.3	15–54	79.2	6.3
Rwanda	2010	14,342	15–49	98.9	15–59	98.2	3.0
Sao Tome and Principe	2009	6,727	15–49	87.5	15–59	70.9	1.5
Tanzania	2008	23,895	15–49	89.5	15–49	79.8	5.7
***West Africa***							
Burkina Faso	2010	21,976	15–49	96.3	15–59	93.7	1.0
Burundi	2010	11,202	15–49	91.8	15–59	88.8	1.4
Cote D’Ivoire	2005	11,970	15–49	79.1	15–49	76.3	4.7
Ghana	2003	13,287	15–49	89.3	15–59	80.0	2.2
Guinea	2005	10,286	15–49	92.5	15–59	88.2	1.5
Liberia	2007	18,048	15–49	87.0	15–49	80.4	1.6
Mali	2006	13,630	15–49	90.7	15–59	83.7	1.3
Niger	2006	13,669	15–49	90.7	15–59	84.2	0.7
Senegal	2011	15,698	15–49	83.7	15–59	76.3	0.7
Sierra Leone	2008	10,934	15–49	87.7	15–59	85.0	1.5

Source: Demographic and Health Surveys (DHS)

* Respondent present on the day of interview, consented to HIV serostatus testing, and tested during their DHS/AIS interview. Percent tested as reported in Demographic patterns of HIV testing uptake in sub-Saharan Africa: DHS Comparative Reports 30. [42] and DHS Country Reports.

** HIV prevalence as available from the HIV/AIDS Survey Indicators Database (accessed at http://hivdata.dhsprogram.com/ on July 17, 2015)

The DHS data reflect nationally representative samples of mostly reproductive aged women and men, with some variability in selected age ranges across the 23 countries. In each country, households were randomly selected for participation and age-eligible men and women completed surveys and were offered HIV testing. Table 1 provides detailed information on age eligibility; in most countries, women aged 15–49 and men 15–59 were eligible. In eight surveys, all households participating in the DHS survey were selected for the HIV test, while in the remaining surveys a random sub-sample of households (usually one-half or one-third) were selected. In most surveys, HIV testing was conducted using dried blood spot (DBS) samples of capillary blood from a finger prick. Details on sample collection and laboratory testing are available elsewhere [40].

We link the results from HIV testing with information on children, their families, and their households collected through the DHS household interviews. We consider the HIV status of all adults who are members of children’s households. As is common in UNICEF reports, children are defined to be those aged 0–17, whenever possible [27]. In five countries, information about parental survival status and residence is collected only for children through age 14, and for these countries, calculations related to orphan status are limited to children through age 14. We report only on co-residence with HIV-infected *adults* because HIV tests in the vast majority of DHS surveys are not administered to children. We adjust our analyses using DHS provided household sample weights. Because these sample weights are at the household level, rather than the level of the individual child, we further adjust the DHS provided weights by the child’s household size.

We calculate the percentage of children living in households with at least one HIV-infected adult from the population of children living in households in which at least one eligible adult aged 18 and older was successfully tested for HIV (i.e., had a positive or negative test). Test participation varies across settings, and is reported in Table 1. Recent assessment of bias in non-response from two-stage and multi-stage estimates suggests that where non-response bias exists, it is in the direction of underestimating HIV prevalence [41]. Consequently, the estimates presented of the percentage of children living in households with infected adults based on the tested population are likely conservative estimates.

Nonetheless, we also calculate a still lower bound estimate that expands the denominator to include all children in households that were *selected* for HIV testing, regardless of whether any adults in the household were present, eligible, or participated in the test. In effect, we assume that all non-tested adults would have tested negative had they been tested. Since children who live in households with adults eligible for testing who are not tested, or who live in households with adults who are not eligible for testing (such as older adults), may well live with an HIV-infected adult, these estimates provide the most conservative benchmark [43].

After estimating the percentage of children living in households with seropositive adults, we investigate overlap between this group and the group of children who are orphaned. The denominator for these calculations is limited to children living in households in which at least one adult 18 or older had a positive or negative HIV test result. Orphan status is determined through responses to questions about parental survival status in DHS household questionnaires. Children who have lost at least one biological parent to death are coded as orphans. When parental status is unknown, children are coded as non-orphans.

In analyses of kin connection, we limit the sample to children who live with at least one seropositive adult. We construct measures that indicate kin connection between each child and the seropositive adults with whom they share a household. We distinguish between mothers, fathers, and other adults, using information from the household schedule on survivorship and residence of biological mothers and fathers. Children can live with more than one adult who has tested positive, and thus separate measures are coded to reflect each relationship. In analysis, we further describe co-residence with HIV-infected adults by summarizing with four mutually exclusive categories: mother but not father, father but not mother, mother and father, or neither mother nor father. In this analysis, co-residence with seropositive adults who are neither mothers nor fathers is possible in every category. Given sample limitations associated with testing, and patterns of non-testing, we emphasize that the estimates presented summarize kin connection only for the sample of children who live in a household with at least one adult with a valid test result, and take into account only tested adults in the household. Other non-tested adults who are seropositive may well live with these children and would not be captured in these figures.

## Results

As shown in Table 2, the percentage of children ages 0–17 living with at least one HIV-infected adult is highest in southern Africa, mid-range in central and east Africa, and lowest in West Africa. In Southern Africa, it ranges from about 14% in Mozambique to 36% in Swaziland. In Central and East Africa, prevalence ranges between 2% in Ethiopia and Sao Tome and Principe to 10% in Kenya. Prevalence is lowest in West Africa, at 1% in Niger and Senegal, but nonetheless, over 7% in Cote D’Ivoire.

**Table 2:** Co-residence of Children 0–17 with HIV-infected adults, by Country.

			Among households with at least one adult *tested* for HIV…		Among all households *selected* for an HIV test….		
Country	Survey year	Adult HIV Prevalence*	N	% children living with at least one HIV+ adult**	N	% children living with adults, but adults not tested	% children living with at least one HIV+ adult***(LOWER BOUND)
***Southern Africa***							
Lesotho	2009	23.0	7,386	33.2	9,987	27.2	24.2
				(32.2–34.3)		(26.3–28.1)	(23.4–25.0)
Malawi	2010	10.6	18,256	15.5	22,788	22.4	12.0
				(15.0–16.0)		(21.9–23.0)	(11.6–12.4)
Mozambique	2009	11.5	12,118	14.2	14,028	14.4	12.2
				(13.6–14.9)		(13.8–15.0)	(11.6–12.7)
Swaziland	2007	25.9	9,219	36.3	11,190	19.1	29.4
				(35.4–37.3)		(18.3–19.8)	(28.6–30.3)
Zambia	2007	14.3	14,317	19.6	19,462	29.1	13.9
				(18.9–20.2)		(28.4–29.7)	(13.4–14.4)
Zimbabwe	2011	15.2	14,384	22.1	20,458	29.7	15.6
				(21.5–22.8)		(29.1–30.3)	(15.1–16.1)
***Central and East Africa***							
Cameroon	2011	4.3	15,492	7.6	17,864	15.3	6.4
				(7.2–8.0)		(14.8–15.8)	(6.1–6.8)
DRC	2007	1.3	11,221	2.6	12,799	13.0	2.3
				(2.3–2.9)		(12.5–13.6)	(2.0–2.5)
Ethiopia	2011	1.5	34,594	2.2	39,538	14.6	1.9
				(2.0–2.3)		(14.3–15.0)	(1.7–2.0)
Kenya	2009	6.3	8,520	10.4	9,615	13.7	9.0
				(9.8–11.1)		(13.0–14.4)	(8.4–9.6)
Rwanda	2010	3.0	13,241	5.0	14,342	10.7	4.5
				(4.6–5.4)		(10.2–11.2)	(4.1–4.8)
Sao Tome and Principe	2009	1.5	5,653	2.3	6,727	19.5	1.9
				(2.0–2.7)		(18.5–20.4)	(1.6–2.2)
Tanzania	2008	5.7	19,957	9.2	23,895	20.5	7.3
				(8.8–9.6)		(19.9–21.0)	(7.0–7.7)
***West Africa***							
Burkina Faso	2010	1.0	19,784	1.7	21,976	11.6	1.5
				(1.6–1.9)		(11.2–12.0)	(1.4–1.7)
Burundi	2010	1.4	10,172	2.4	11,202	11.6	2.1
				(2.1–2.7)		(11.0–12.2)	(1.9–2.4)
Cote D’Ivoire	2005	4.7	9,247	7.5	11,970	27.8	5.4
				(6.9–8.0)		(27.0–28.6)	(5.0–5.8)
Ghana	2003	2.2	10,525	3.0	13,287	23.8	2.3
				(2.6–3.3)		(23.1–24.6)	(2.0–2.5)
Guinea	2005	1.5	8,587	2.4	10,286	19.1	1.9
				(2.0–2.7)		(18.3–19.8)	(1.6–2.2)
Liberia	2007	1.6	14,455	1.8	18,048	20.4	1.4
				(1.6–2.0)		(19.9–21.0)	(1.3–1.6)
Mali	2006	1.3	11,458	1.9	13,630	17.9	1.6
				(1.7–2.2)		(17.3–18.5)	(1.4–1.8)
Niger	2006	0.7	11,547	1.0	13,669	18.1	0.8
				(0.8–1.2)		(17.5–18.8)	(0.7–1.0)
Senegal	2011	0.7	13,569	1.3	15,698	15.2	1.1
				(1.2–1.5)		(14.6–15.7)	(1.0–1.3)
Sierra Leone	2008	1.5	8,579	2.0	10,934	23.0	1.5
				(1.7–2.3)		(22.2–23.8)	(1.3–1.8)

*Source*: Demographic and Health Surveys (DHS)

Note: The measures use household weights provided by the DHS as well as weights for household size.

* DHS estimates, HIV prevalence among adults 15–49.

** Denominator is limited to children 0–17 living in households in which at least one adult aged 18+ had a positive or negative HIV test result.

*** Denominator includes all children 0–17 living in households selected for HIV testing. The lower bound estimate assumes that in households in which no adult was tested, if all eligible adults were tested none would test positive.

**Fig 1:**
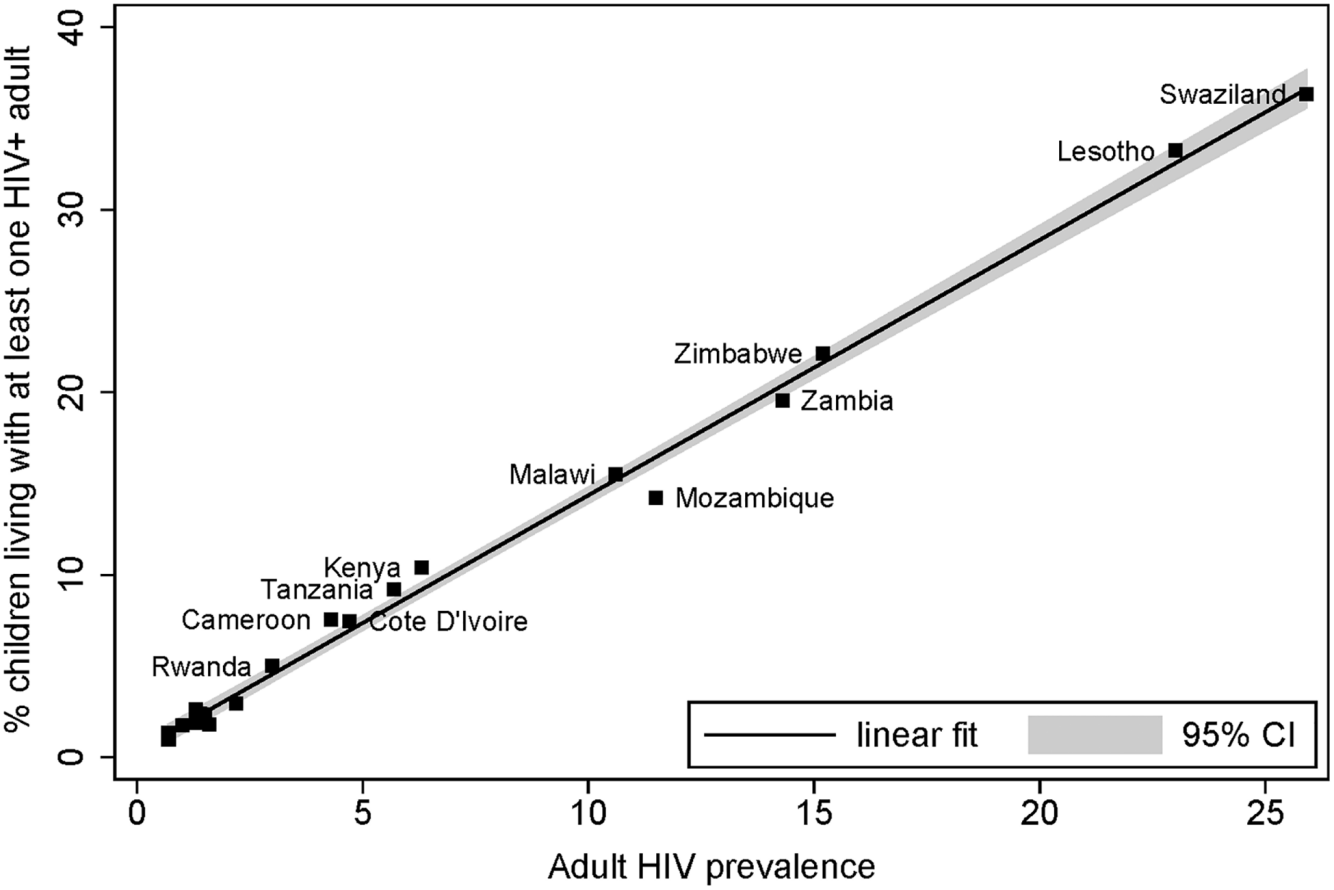
Co-residence of Children 0–17 with HIV-infected Adults and Adult HIV Prevalence (15–49), by Country. *Source*: Demographic and Health Surveys (DHS).

Fig 1 plots the percentage of children living in a household with at least one HIV-infected adult against adult HIV prevalence for each country. Although the timing and nature of the HIV/AIDS epidemic varies across countries, as do programmatic responses and family organization, the plot suggests that children’s likelihood of living in households with HIV-infected adults tracks HIV prevalence closely, such that the relationship is near linear.

While only a small fraction of children live in households without an adult (less than 1.6% in every country, not shown), substantial fractions of children live in households in which no adults, or not all adults, were tested. While testing rates are high in most of the DHS/AIS surveys (Table 1), they are based on those eligible for testing. Further, in some cases, both women and men are selected for interview and testing, but only one may be tested. In other cases, one or more adults in the household may fall out of the eligible age range for testing. In cases where one adult tests negative, non-tested adults could be positive. Accordingly, in the final two columns of Table 2, we present a lower bound and upper bound estimate that takes into account the population not tested. The lower bound estimate is the percentage of children living in a household with at least one HIV-infected adult, under the assumption that *all* non-tested adults are *not* infected. The lower bound calculations affect the estimates most in Southern Africa, where both HIV prevalence and the percentage of those not tested is high. Still, even under the assumption that all non-tested adults are negative, the percentage of children living with HIV-infected adults is close to 30% in Swaziland, and between 12% and 24% across the remaining five countries in Southern Africa.

In Table 3 we explore the degree to which the population of children living with HIV-infected adults is distinct from that of children who are orphans. Results indicate that most are not orphans. For example, while 33% of children in Lesotho in 2010 were living with an HIV-infected adult, only 10% were both living with an HIV-infected adult *and* orphaned. Similarly, in Zimbabwe, while 22% of children lived with an HIV-infected adult, only 6% both lived with an HIV-infected adult and were orphaned. Considering both adult mortality and infection reveals the extensive reach of the epidemic into children’s lives. In Lesotho and Swaziland, where about 70% of orphans are estimated to be orphaned due to AIDS, a staggering 50% of children are either orphans or live in a household with an HIV-infected adult [1].

**Table 3:** Co-residence of Children 0–17* with HIV-infected Adults and Orphan Status, by Country**.

Country	Survey year	N	Adult HIV Prevalence***	% living with HIV+ adult, not orphaned	% living with HIV+ adult, orphaned	% not living with HIV+ adult, orphaned	% not living with HIV+ adult, not orphaned
***Southern Africa***							
Lesotho	2009	7,386	23.0	23.2	10.0	16.3	50.4
				(22.3–24.2)	(9.3–10.7)	(15.5–17.2)	(49.3–51.6)
Malawi	2010	18,256	10.6	12.3	3.2	7.1	77.4
				(11.8–12.7)	(3.0–3.5)	(6.7–7.5)	(76.8–78.0)
Mozambique	2009	12,118	11.5	11.4	2.8	9.2	76.5
				(10.9–12.0)	(2.5–3.1)	(8.7–9.7)	(75.8–77.3)
Swaziland	2007	9,219	25.9	26.5	9.8	13.7	49.9
				(25.6–27.4)	(9.2–10.4)	(13.0–14.5)	(48.9–50.9)
Zambia	2007	14,317	14.3	15.7	3.9	7.6	72.8
				(15.1–16.3)	(3.6–4.2)	(7.2–8.1)	(72.1–73.5)
Zimbabwe	2011	14,384	15.2	16.0	6.1	11.5	66.3
				(15.4–16.6)	(5.7–6.5)	(11.0–12.1)	(65.6–67.1)
***Central and East Africa***							
Cameroon	2011	15,492	4.3	6.6	1.0	8.5	84.0
				(6.2–7.0)	(0.8–1.1)	(8.0–8.9)	(83.4–84.6)
DRC	2007	11,221	1.3	2.2	0.4	8.2	89.2
				(2.0–2.5)	(0.3–0.5)	(7.7–8.7)	(88.7–89.8)
Ethiopia	2011	34,594	1.5	1.7	0.5	8.4	89.4
				(1.5–1.8)	(0.4–0.6)	(8.2–8.7)	(89.1–89.7)
Kenya****	2003	6,429	6.3	8.5	2.3	8.8	80.3
				(7.9–9.2)	(2.0–2.7)	(8.1–9.5)	(79.4–81.3)
Rwanda	2010	13,241	3.0	3.7	1.4	12.2	82.9
				(3.3–4.0)	(1.2–1.5)	(11.6–12.7)	(82.2–83.5)
Sao Tome and	2009	5,653	1.5	2.3	0.1	3.6	94.1
Principe				(1.9–2.7)	(0.0–0.1)	(3.1–4.0)	(93.5–94.7)
Tanzania	2008	19,957	5.7	7.5	1.7	7.3	83.5
				(7.1–7.9)	(1.5–1.9)	(7.0–7.7)	(83.0–84.0)
***West Africa***							
Burkina Faso	2010	18,106	1.0	1.6	0.1	3.6	94.7
				(1.4–1.8)	(0.1–0.2)	(3.3–3.9)	(94.4–95.1)
Burundi	2010	10,172	1.4	2.0	0.4	11.8	85.8
				(1.7–2.2)	(0.3–0.6)	(11.2–12.4)	(85.2–86.5)
Cote D’Ivoire	2005	9,247	4.7	6.6	0.9	6.1	86.4
				(6.1–7.1)	(0.7–1.1)	(5.6–6.6)	(85.7–87.1)
Ghana	2003	10,525	2.2	2.7	0.3	4.9	92.2
				(2.4–3.0)	(0.2–0.4)	(4.5–5.3)	(91.7–92.7)
Guinea	2005	8,035	1.5	1.8	0.5	5.9	91.8
				(1.5–2.1)	(0.3–0.6)	(5.4–6.4)	(91.2–92.4)
Liberia	2007	14,455	1.6	1.6	0.2	6.1	92.2
				(1.4–1.8)	(0.1–0.3)	(5.7–6.4)	(91.7–92.6)
Mali	2006	10,748	1.3	1.8	0.2	5.5	92.6
				(1.5–2.0)	(0.1–0.2)	(5.1–5.9)	(92.1–93.1)
Niger	2006	10,970	0.7	0.8	0.2	4.4	94.6
				(0.6–1.0)	(0.1–0.2)	(4.0–4.8)	(94.2–95.1)
Senegal	2011	13,569	0.7	1.3	0.1	6.4	92.3
				(1.1–1.5)	(0.0–0.1)	(5.6–6.8)	(91.8–92.7)
Sierra Leone	2008	8,579	1.5	1.8	0.2	8.6	89.4
				(1.5–2.1)	(0.1–0.3)	(8.1–9.2)	(88.7–90.0)

*Source*: Demographic and Health Survey (DHS)

Note: The measures use household weights provided by the DHS as well as weights for household size.

* The statistics for Kenya, Burkina Faso, Guinea, Mali, and Niger exclude children 15–17 because parental survival status is not available for this age group.

** In columns 5–8, the denominator is limited to children living in households in which at least one adult 18+ had a positive or negative HIV test result.

*** DHS estimates, HIV prevalence among adults 15–49.

**** Data from the Kenya 2003 survey are used in the current table because information on parental survival status was not collected in the 2009 survey.

**Fig 2:**
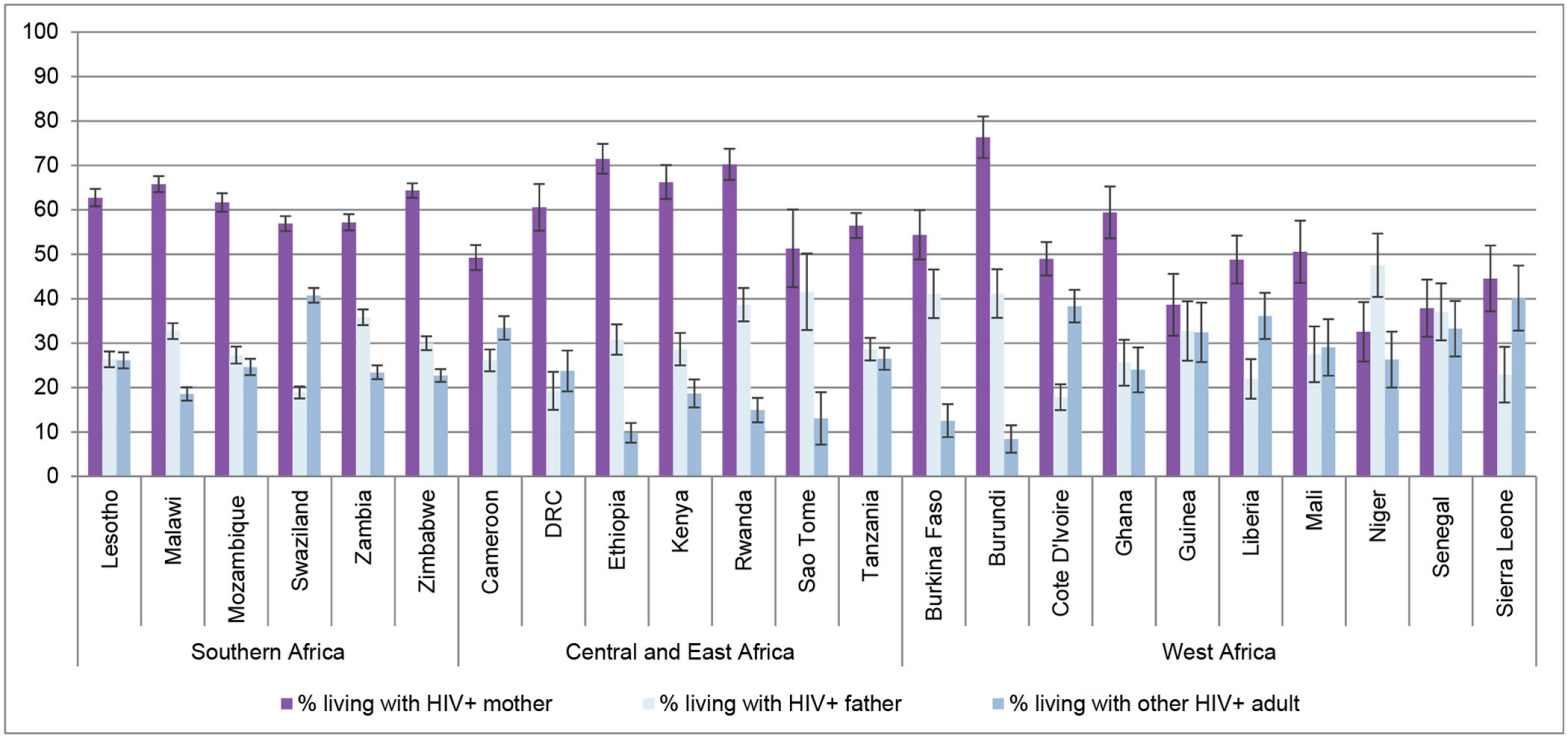
Among Children 0–17* Living with at Least One HIV-infected Adult, Percent Living with HIV-infected Mothers, Fathers, and Others, by Country. *Source*: Demographic and Health Surveys (DHS). * The statistics for Kenya, Burkina Faso, Guinea, Mali, and Niger exclude children 15–17 because information on parental co-residence was not collected for this age group. ** Data from the Kenya 2003 survey are used because information on parental co-residence was not collected in the 2009 survey. *Note*: The measures use household weights provided by the DHS as well as weights for household size.

Next, Fig 2 shows the relationship between children and the HIV-infected adults in their households, as reflected in the tested sample. In the majority of cases, children who are living with an HIV-infected adult in the household are living with at least one HIV-infected parent, most often an HIV-infected mother. In Southern Africa, where HIV prevalence is highest, over two-thirds, and usually about 80%, of children who live in households with an HIV-infected adult, live with a parent who is HIV-infected. These percentages are similar in Central, East, and West Africa. Despite the preponderance of co-residence with HIV-infected parents, it is nonetheless important to note that in most countries over 20%, and sometimes over one-third, of children live in a household with at least one HIV-infected adult who is not a parent.

Finally, Table 4 presents the extent to which children live with an HIV-infected mother, father, both, or neither, among the sample of children who live with at least one HIV-infected adult in their household. While it is most common for children to live with an HIV-infected mother only, a substantial fraction, more than 10% in every country, live in a household in which both their mother and father is infected. We emphasize, however, that children’s living arrangements vary significantly across these settings, and these statistics reflect the relationships of the tested, HIV-infected adults in the household to the children; parents who are not HIV-infected may be present or absent, and some who are present may not be tested. Nonetheless, these data provide a glimpse into the nature of relationships among tested adults and children as available in the DHS.

**Table 4:** Among Children 0–17* Living with HIV-infected Adults, Percent Living with HIV-infected Parents.

Country	Survey year	N	% living with HIV+ mother and not HIV+ father	% living with HIV+ father and not HIV+ mother	% living with HIV+ father and HIV+ mother	% living with neither HIV+ father nor HIV+ mother
***Southern Africa***						
Lesotho	2009	2,340	51.6	15.2	11.1	22.1
			(49.6–53.6)	(13.8–16.7)	(9.9–12.4)	(20.4–23.7)
Malawi	2010	2,624	51.7	18.6	14.1	15.6
			(49.8–53.6)	(17.1–20.1)	(12.8–15.4)	(14.2–17.0)
Mozambique	2009	2,096	51.5	17.2	10.2	21.2
			(49.4–53.7)	(15.5–18.8)	(8.9–11.5)	(19.4–23.0)
Swaziland	2007	3,349	48.0	10.0	8.9	33.1
			(46.3–49.7)	(9.0–11.0)	(7.9–9.8)	(31.5–34.7)
Zambia	2007	2,784	44.6	23.3	12.5	19.6
			(42.8–46.5)	(21.7–24.9)	(11.3–13.8)	(18.1–21.0)
Zimbabwe	2011	3,308	50.6	16.2	13.7	19.5
			(48.9–52.3)	(15.0–17.5)	(12.6–14.9)	(18.1–20.8)
***Central and East Africa***						
Cameroon	2011	1,232	43.3	20.2	6.0	30.6
			(40.5–46.0)	(17.9–22.4)	(4.7–7.3)	(28.0–33.2)
DRC	2007	333	57.3	16.0	3.2	23.4
			(52.0–62.7)	(12.1–20.0)	(1.3–5.1)	(18.8–28.0)
Ethiopia	2011	696	60.8	20.1	10.7	8.4
			(57.1–64.4)	(17.1–23.1)	(8.4–13.0)	(6.4–10.5)
Kenya**	2009	596	55.0	17.4	11.2	16.3
			(51.0–59.0)	(14.4–20.5)	(8.7–13.8)	(13.4–19.3)
Rwanda	2010	657	48.9	17.4	21.3	12.4
			(45.1–52.8)	(14.5–20.3)	(18.1–24.4)	(9.9–15.0)
Sao Tome and	2009	129	45.4	35.6	5.9	13.0
Principe			(36.7–54.1)	(27.3–44.0)	(1.8–10.0)	(7.2–18.9)
Tanzania	2008	1,207	49.1	21.2	7.4	22.3
			(46.3–51.9)	(18.9–23.6)	(5.9–8.9)	(20.0–24.7)
***West Africa***						
Burkina Faso	2010	312	47.1	33.9	7.2	11.8
			(41.6–52.7)	(28.6–39.1)	(4.3–10.1)	(8.2–15.4)
Burundi	2010	317	50.4	15.3	25.9	8.4
			(44.9–56.0)	(11.3–19.2)	(21.1–30.8)	(5.3–11.5)
Cote D’Ivoire	2005	680	45.8	14.6	3.2	36.4
			(42.0–49.5)	(12.0–17.3)	(1.9–4.5)	(32.8–40.0)
Ghana	2003	277	51.7	17.9	7.7	22.7
			(45.8–57.7)	(13.4–22.4)	(4.5–10.8)	(17.7–27.7)
Guinea	2005	192	34.9	29.0	3.7	32.4
			(28.1–41.7)	(22.5–35.5)	(1.0–6.4)	(25.7–39.1)
Liberia	2007	335	44.7	17.9	4.1	33.4
			(39.4–50.1)	(13.7–22.0)	(2.0–6.2)	(28.3–38.4)
Mali	2006	198	43.5	20.4	7.1	29.0
			(36.5–50.5)	(14.8–26.1)	(3.5–10.7)	(22.6–35.4)
Niger	2006	192	26.8	41.8	5.7	25.7
			(20.5–33.2)	(34.8–48.9)	(2.4–9.0)	(19.4–31.9)
Senegal	2011	222	31.0	30.2	6.9	32.0
			(24.8–37.1)	(24.1–36.2)	(3.5–10.3)	(25.8–38.2)
Sierra Leone	2008	176	37.6	15.9	7.0	39.5
			(30.4–44.8)	(10.5–21.4)	(3.2–10.8)	(32.3–46.8)

Source: Demographic and Health Survey (DHS)

Note: The measures use household weights provided by the DHS as well as weights for household size.

* The statistics for Kenya, Burkina Faso, Guinea, Mali, and Niger exclude children 15–17 because information on parental co-residence is not available for this age group.

** Data from the Kenya 2003 survey are used because information on parental co-residence was not collected in the 2009 survey.

## Discussion

The estimates we present from 23 countries across sub-Saharan Africa demonstrate that the population of children living in a household with an HIV-infected adult is large where HIV prevalence is high, and that it is somewhat distinct from the orphan population. The majority of children living in households with HIV-infected adults, at least as represented in the DHS samples, live with parents, often mothers, who are HIV-infected. Nonetheless, a non-trivial share of children lives with an HIV-infected adult who is not a parent.

As Richter and colleagues have observed, children and families have been severely neglected in response to the HIV/AIDS epidemic [44]. We suggest that children living in households with HIV-infected adults are distinct from other HIV-affected children, and call for an increase in targeted attention to their needs, at the same time emphasizing that their lives are enriched and enhanced because they share their households with these very same adults. Thus, the challenge and opportunity is to design effective family outreach that both affirms the family and supports healthy child development. We observe that current efforts to develop and monitor the care cascade—which brings heightened attention to the sustained, effective treatment of HIV-infected individuals—brings needed resources that will support the health of HIV-infected individuals and their families.

Indeed, family-based care has been recognized as critical to effective AIDS response, although targeted attention to the population of children living with HIV-infected adults is limited. For example, the PEPFAR blueprint for creating an AIDS-free generation [35] suggested that programmatic activities include family outreach to HIV-infected mothers who have participated in PMTCT programs. This outreach is focused on extending HIV testing and counseling, prevention, and ART services to partners and families; providing mothers with counseling and support related to infant feeding and infant care; and providing links to OVC social services. Similarly, Heymann, Clark, and Brewer [45] have advocated for a “preventing family illness and death” (PFID) approach to protecting families from HIV/AIDS. This approach focuses on prevention of adult infection, treatment of children’s infected parents and caregivers, and orphan care. Deepening outreach to children co-residing with HIV-infected adults in ways that recognize their specific vulnerabilities could enhance ongoing and planned strategies geared toward maintaining wellness in families affected by HIV/AIDS [14, 17]. It would also be consistent with approaches that recognize risk in a social determinants framework, which situates HIV/AIDS vulnerability in family and other social and environmental contexts [46–50].

Fortunately, programs that provide support for vulnerable and AIDS-affected children exist. Further, they can be associated with positive outcomes in children. Many take the form of cash transfers to poor and vulnerable, often AIDS-affected, households, thus targeting a broad group of children and families in need [51–53]. Given the growing care deficit, and the physical and mental health challenges that may beset children in high HIV prevalence areas, such interventions are increasingly evaluated for their effects on children’s physical and mental health. A recent prospective study in Kenya suggested that orphans and vulnerable adolescents in cash transfer households reported better psychological health than those in non-cash transfer households [53]. New interventions are also experimenting with supplementing cash transfers with other forms of support. A randomized control trial in South Africa suggests that “cash plus care” interventions, and specifically the receipt of both economic and psychosocial support, can be associated with reduced HIV-risk behaviors among adolescents [54]. In Botswana, local support programs include caregiving support for HIV-affected families, and the need for such care has garnered significant attention and interest in expanding quality programming [55]. Together, these efforts, spread across numerous settings, suggest an increase in targeted interventions is likely, although the financial and logistical challenges to such programs are many [55].

We acknowledge several key limitations of this study. First, the estimates we present include only countries with available DHS data on HIV testing and parental survival. Some countries with high HIV prevalence (such as South Africa) or well-known HIV/AIDS programs (such as Uganda) are thus not included. Second, our estimates are biased downward because the DHS limits testing to adults of reproductive age in most countries, excluding adults over the age of 50, some of whom are likely to be infected [43]. Substantial levels of non-response among adults eligible for testing render our results among households with adults tested still more conservative, for reasons explained above. In addition, in using cross-sectional data, we present a snapshot at one point in time. If appropriate cross-national data existed to allow us to estimate the percentage of children who ever lived in a household with an HIV-infected adult before age 18, it is likely that many more children would be affected. Finally, we do not know whether the adults know their status or are ill as a result of HIV infection. The implications for children will depend on adults knowing their status and accessing recommended treatment.

Notably, we call for attention to the population of children co-residing with HIV-infected adults in sub-Saharan Africa at the same time others urge programs to move away from a focus on orphans and AIDS-affected children and towards a more general emphasis on vulnerable children [56]. We propose that these two suggestions need not be at odds, and both should be pursued. All vulnerable children, including all children living in poverty, should be targeted in efforts to promote child well-being. However, children living with adults who are HIV-infected may have distinct vulnerabilities. Not only may they be at increased risk of direct exposure to opportunistic infections, but they may well experience a cascade of challenges associated with HIV, including stigma and disrupted social networks, diversion of resources and attention, and increases in poverty, all of which may influence their mental and physical health [26, 44, 57–59]. Notably, this cascade is characterized by overlaps which can intensify the challenges. For example, increased exposure to infection is a specific vulnerability that may be exacerbated by poverty, or a disruption in social networks, or limited child care that results from competing household demands [57, 60]. Further, community context can amplify these challenges; children living in households with HIV-infected adults may be particularly vulnerable to the growing care deficit in high prevalence settings or they may live in communities where AIDS is highly stigmatized [61].

In closing, more relevant to the suggestion that HIV-exposed children receive targeted intervention is the practical challenge of identifying such children. Family outreach upon the birth of a child, during testing and counseling, or during treatment, may offer opportunity in this regard. However, in settings with the highest HIV prevalence, where up to one-third of children live in households with HIV-infected adults, in the absence of individual identification and intervention, targeted public health messages promoting children’s health in areas of a generalized HIV epidemic would be of potential benefit to all children and families.
